# A new perspective from time use research on the effects of social restrictions on COVID-19 behavioral infection risk

**DOI:** 10.1371/journal.pone.0245551

**Published:** 2021-02-10

**Authors:** Jonathan Gershuny, Oriel Sullivan, Almudena Sevilla, Marga Vega-Rapun, Francesca Foliano, Juana Lamote de Grignon, Teresa Harms, Pierre Walthery

**Affiliations:** Centre for Time Use Research, Social Research Institute, University College London, London, United Kingdom; London School of Hygiene and Tropical Medicine, UNITED KINGDOM

## Abstract

We present findings from three waves of a population-representative, UK time-use diary survey conducted both pre- and in real time during full ‘lockdown’, and again following the easing of social restrictions. We used an innovative online diary instrument that has proved both reliable and quick-to-field. Combining diary information on activity, location, and co-presence to estimate infection risks associated with daily behavior, we show clear changes in risk-associated behavior between the pre, full-lockdown and post full-lockdown periods. We document a shift from more to less risky daily behavior patterns (combinations of activity/location/co-presence categories) between the pre-pandemic pattern and full lockdown in May/June 2020, followed by a reversion (although not a complete reversal) of those patterns in August 2020 following the end of the first lockdown. Because, in general, a populations’ time use changes relatively slowly, the behavioral changes revealed may be interpreted as a consequence of the UK COVID-19 lockdown social restrictions and their subsequent relaxation.

## Introduction

Governments around the world are urgently redesigning social distancing measures as they assess the trade-offs between economic and psychological distress and the need to avoid resurgences in COVID-19. At a time when there is still limited capacity in respect both of immunization and track-trace technology, governments must continue to rely on changes in people’s daily behaviors to contain the virus. In this paper we introduce a new time use based approach enabling policy-makers to assess and quantify changes in daily behaviors following the imposition of social restrictions, and the impact they are likely to have on overall behavioral-associated risks. Using an online time-use diary survey first deployed in the UK in 2016, then conducted in real time during the period of full lockdown (late May 2020) and again following the easing of social restrictions in July 2020, we provide an overview of changing behaviors associated with infection risk both prior to, and during, recent phases of social restrictions.

Many aspects of the COVID-19 pandemic are still poorly understood. Of the main approaches to modelling the heterogeneity of infection, demographic approaches have focused on a variety of individual risk-related characteristics—age, sex and ethnicity in particular [1–3]. But these demographic characteristics, although they are shown to be important discriminators in terms of infection risk, are less proximate than are the daily patterns of behavior which underlie the actual routes of infection. It is clear that the effective means of transmission of infection is *contact*: close interaction of an uninfected individual with an infected individual or location [4]. Contact-based instruments, derived particularly from ‘Polymod’ [5, 6], record the actual physical proximity of contact (such as the speaking/touching/distancing distinction). Such surveys ask respondents who they met during specific periods (e.g. ‘yesterday’ or ‘between these times’), or while they were in a defined area at a particular time of the day, and what the extent of their physical contact was with others during that time [6–8]. This type of information has been the main source for the formal modelling of infection spread; for example, contact-based surveys showed substantial decreases in physical contact following the imposition of first lockdown in the UK [8]. However, contact-based surveys have often not provided much information on the types of locations in which contacts occurred, or on the likelihood that respondents were surrounded by people other than those they describe as ‘contacts’ (e.g. on a train, or in the cinema), or about time spent in activities/locations during which respondents did not report being in contact with specific others.

Time use diary data complements contact-based information by providing rich contextual information on the entire daily sequence of a population’s activities, location and co-presence, enabling a complete picture of the types of daily behavior that characterise the different phases of social restrictions to be constructed. It is regarded as the ‘gold standard’ when it comes to the collection of information on population activity distributions and sequences [9]. Major COVID-19 related UK social surveys conducted over the course of the pandemic have not collected time-use diaries [10], while the UK Office of National Statistics collected a pilot online time-use diary only once during the pandemic, in March-April 2020 [11], without linking the results to behavioral infection risk. However, when time use diary-derived patterns of behavior are linked to infection risk (through assessment of the ‘riskiness’ of certain combinations of activity, location and co-presence), this enables the identification of those changes in behavior patterns which are likely to contribute to subsequent changes in infection rates.

While contact-based studies showed substantial decreases in physical contact following the imposition of first lockdown in the UK, our three-wave record of changing behavior between the pre-pandemic and first lockdown period reveals which actual behaviors (activities and locations and co-presences) were substituted under lockdown for pre-lockdown behavior (for example, lower-risk time at home substituting for higher-risk time at the workplace). Subsequently, in August 2020, we observe the modification of behavior in the direction of more time again being spent in more risky behaviors (associated with the easing of social restrictions), leading in turn, via increasing infection rates, to the imposition of a second lockdown.

Time-use diary data has been extensively deployed over many decades to understand trends in daily behavior [9, 12, 13]. Time-use diary surveys provide nationally-representative samples of comprehensive, continuously-registered, records about the activities of daily life, including detail on their location and social context, through every 10 minutes of a 24 hour (or longer) period. Comparisons with objective activity measures (worn cameras, accelerometers) suggest that time-use diaries provide reliable and unbiased records of activity sequences and durations [14]. Deploying time-use diary evidence in this context has been done before [15–17], but not in respect of combining multiple diary fields (activities, locations, co-presence and durations) to estimate behavior-related infection risks, and not to report changing behavioral-related infection risks at successive surveys reflecting periods of changing social restrictions.

## Materials and methods

In 2016 the Centre for Time Use Research (together with the Dynata survey agency and the Trajectory Partnership research consultancy), developed a new online Click and Drag Diary Instrument (CaDDI), collecting population-representative (quota sample) time use diary data from Dynata’s large existing market research panel across 9 developed countries including the UK and the USA [18]. We fielded the same instrument using the same UK research panel in May-June 2020—at the peak period of lockdown, providing a real-time comparison with 2016 behavior, and again in late August 2020 following the relaxation of social restrictions in July 2020.

The quota sample was selected from the Dynata research panel to represent the UK population aged 18+. Research panel members were recruited separately for each wave and accepted up to the point the quota limits were reached. Quotas were set to represent the UK population in 2016 according to sex, age group, region and social grade. Response days (1–3 per respondent) were randomly allocated to respondents, to include one weekday and one weekend day where there was more than one diary completed (most respondents completed 2 or 3 diaries). Diaries were first reweighted for analysis to ensure a correct distribution of days of the week by sex and age group. For each survey wave we took 6 (broadly 10-year) age-groups, the two sex-groups and each day of the week, adjusting the achieved sample for each of the resulting (3*6*2*7) cells to give the appropriate representation of each day, then normalizing to retain the original overall sample numbers. The survey waves provided 1011 diary days in 2016, 1007 in May/June 2020, and 987 in August 2020.

Table 1 shows the distribution of diary days according to the nationally-representative quota target specifications for sex, age, region and social group in 2016. In general there is a close fit between quota targets and achieved diary numbers across the survey waves, with three exceptions: there is an under-representation of the 65+ age group in the two recent surveys, a lesser under-representation among the 18–24 group in the August 2020 survey only, and an under-representation of social grade C2 in the two 2020 survey waves (although quota specifications were met for grades D+E). In the case of social group the difference may reflect a move from C2 employment into unemployment in the population (and also among panel members) as a direct effect of the pandemic. With respect to age it is possible that the changes in the quota representation across waves represents a response bias due to pandemic conditions, with both older and younger age groups having been seriously impacted by social restrictions. We therefore also reweighted the 2020 samples according to the 2016 quota distribution for age group 65+ (normalizing again for achieved sample sizes). While total sample size allowed us to adjust the achieved sample to compensate for under-representation of the older age group cells in the 2020 waves, further sub-division by social group produced too many vacant cells. The fact that quotas were met for social grades D+E, however, means that the social grade distribution did include some representation from the lower end of the scale. We use the reweighted samples for our analyses.

**Table 1:** Sample quota groups, target percentages and achieved % of diaries: CaDDI 3-wave data.

Quota groups	Quota target	Unweighted diaries %		
		2016	May-20	Aug-20
**Sex**		**%**	**%**	**%**
Male	49%	51	52	50
Female	51%	49	48	50
**Age group**				
18–24	13%	13	11	7
25–34	16%	13	22	20
35–44	19%	17	22	25
45–54	17%	18	23	24
55–64	15%	18	17	21
65+	20%	21	6	2
**Region**				
London	13%	10	15	13
Yorks & Humber	9%	9	9	9
East Midlands	7%	8	9	8
East Anglia	9%	10	10	9
South East	14%	14	9	15
South West	9%	9	8	8
West Midlands	9%	8	11	8
North West	11%	12	11	9
Scotland	8%	9	8	8
Wales	5%	5	4	4
N Ireland	3%	2	2	1
North East	4%	5	4	5
**Social Group**				
AB	26%	27	36	35
C1	29%	31	32	34
C2	21%	20	10	9
DE	24%	23	23	23
**N of diaries = 100%**		1011	1007	987

The CaDDI survey proved cheap to administer and relatively non-burdensome for respondents, with a mean 15-minute, median 12-minute, collection time for the first diary of the first 2020 wave (i.e. before respondents had a chance to familiarize themselves with the diary). New waves of data collection using the same methodology are quick to commission (both 2020 survey waves took less than a fortnight from first consideration to enter the field). Response quality was comparable to other on-line diaries, with a mean of 19 episodes recorded each day across all three waves. Due to the sampling design (using existing research panel members), there were unusually low levels of missing primary activity, location and co-presence data. Consequently, our behavioral risk variable has an average of only 14 minutes (out of 1440) missing or unallocated per day in 2016, 13 minutes/day missing in May-June 2020, and 12 minutes/day missing in August 2020.

We analyze the anonymized survey data administered by Dynata. The 2020 waves of our survey were approved by the UCL Institute of Education Research Ethics Committee under the Social Science/Market Research Society protocol. The first wave was approved in 2015 by the University of Oxford Department of Sociology Ethics Committee.

Taking cognisance of the epidemiological literature on contact risks, the estimates of risk levels we used were based on combinations of activity type, location and co-presence. We combined records of *activity* (36 categories), *co-presence* (7 categories, with up to four categories recorded simultaneously), *location* (3 categories), and duration, to estimate different levels of risk of infection for each 10-minute period through the day. The literature on COVID-19 infection transmission considers time at home alone or with members of the same household as lowest-risk, with the main focus for transmission being contact with non-household members inside or outside the home. The virus is more likely to be transmitted indoors, in crowds, and through personal contact of over 15 minutes [19, 20]. Our risk estimates located each activity/ location/ co-presence/duration combination accordingly in one of five risk-level categories ordered from low to high.

Our estimates of riskiness of location and co-presence status vary according to the activity—sometimes reflecting the nature of the activity itself (e.g. cinema implies the presence of other, non-household, individuals), and sometimes influenced by its characteristic location (e.g. indoors enclosed, vs open-air). The multiple diary fields for each episode assist the risk-assignment process. For example, co-presence information may be supplemented from the activity fields, so ‘using public transport’ can be taken to imply current or recent presence of other, non-household, individuals, while ‘childcare’ implies presence of children. Registered activity may directly indicate risk: ‘jogging’, for example, implying a low risk open-air location, ‘at the cinema’, an enclosed space.

The resulting estimates of risk accompanying the activity/co-presence/location combinations are shown in Table 2. Activities are shown grouped into categories that have similar patterns of risk attribution across the location and co-presence fields. The main two columns show the ‘in home’ and ‘away from home’ location categories, each grouped into two co-presence categories reflecting lower and higher risk respectively: alone or together with household members; and together with other, non-household members.

**Table 2:** Risk-level assignments, by activity, location and co-presence categories.

	__________Assigned risk level__________			
	_____At home____		Away from home	
	Alone/HH	Non-HH	Alone/HH	Non-HH
	(Column 0)	.(Column 1)	(Column 2)	(Column 3)
**Activity number and description**				
1 Sleeping	1	4	2	5
2 Resting	1	4	2	5
3 Washing, dressing	1	4	2	5
5 Preparing food, cooking, washing up	1	4	2	5
6 Cleaning tidying house	1	4	2	5
7 Clothes washing, mending, sewing	1	4	2	5
8 Maintenance of house, diy, gardening	1	4	2	5
21 Caring for own children	1	4	3	5
23 Help, caring for co-resident adults	1	4	3	5
27 Watching tv, video, dvd, radio, other music	1	4	3	5
28 Reading including e-books	1	4	3	5
29 Playing sports, exercise	1	4	3	5
32 Playing computer games	1	4	3	5
33 Spending time with friends, family	1	4	3	5
34 Telephone, text, email, networking, letters	1	4	3	5
36 Hobbies	1	4	3	5
11 Travelling: walking, jogging			2	3
12 Travelling: cycle			2	3
31 Walking, dog walking			2	3
13 Travelling: car			2	5
14 Travelling: bus, tram			5	5
15 Travelling: train, tube			5	5
16 Travelling: other			5	5
30 Going out to eat, drink eg pub, restaurant			5	5
35 Cinema, theatre, sports, cultural event			5	5
4 Eating, drinking, meal, at home	1	4	4	5
22 Caring for other children	3	4	3	5
24 Help, caring for noncoresid adults unpaid	3	4	3	5
9 Services: Doctor, dentist, hairdresser	3	4	3	5
26 Shopping, bank etc including internet	1	4	5	5
10 Church, temple, mosque, synagogue, prayer	1	4	5	5
17 Paid work including at home	1	4	5	5
18 Formal education	1	4	5	5
19 Recreational courses, study	1	4	5	5
20 Voluntary work for club, organisation	1	4	3	5
25 Work, study break	1	4	2	5
37 Other not listed (excluded)				

Following the suggestion of de Cao et al. [19], we then took account of the duration of events, assigning activities lasting only one 10-minute timeslot to the lowest risk level (level 1). This leads to only a small number of changes in our risk assignments. S1 Table of the Supporting Information provides a look-up table setting out the detailed composition of the nine activity/copresence/location categories, based on the activity number and column number information shown in Table 2.

Although our findings are based on a quota sample rather than on a standard random sample, we use regression analyses to provide suggestive estimates of the significance of the differences between the mean time spent in the lowest and highest risk categories across the survey waves. We ran two models, one for each of the two risk categories. The dependent variables in these models are mean time spent in each respective risk category (1 and 5), and the independent is a survey wave dummy. June 2020 –i.e. full lockdown—was the reference category. Robust standard errors were clustered at the level of the individual to take account of the differing numbers of diary days per respondent.

## Results

**Fig 1:**
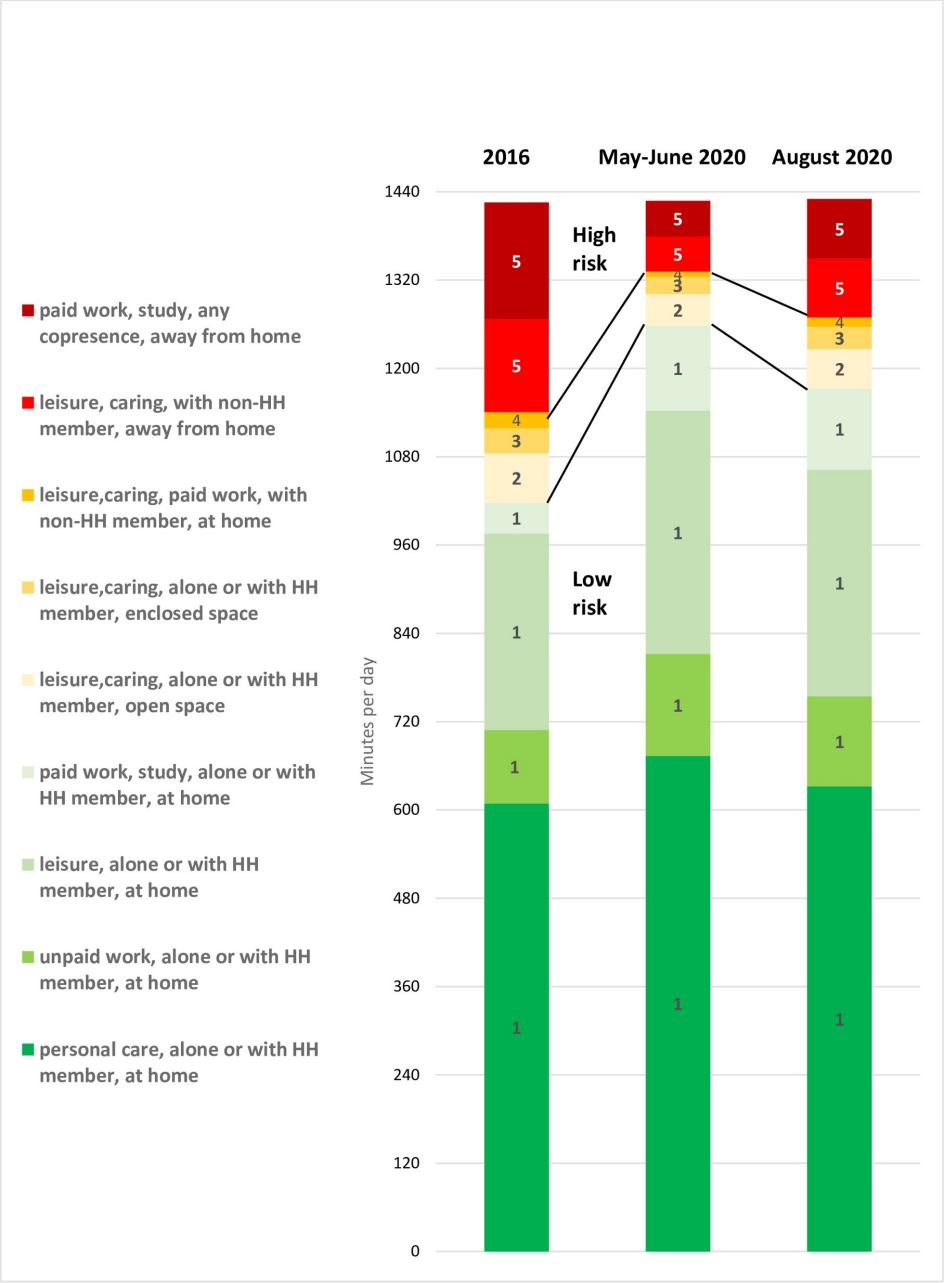
Mean minutes per day spent at 5 different behavioral risk levels, grouped by activity/copresence/location combinations: UK population pre- first lockdown, mid- first lockdown and following lockdown.

Fig 1 presents a view of change in behavior related to infection risk between the 2016 survey, that conducted in May-June 2020, and that in late August 2020. It shows change in mean minutes per day in the different combined activity/location/co-presence categories in the UK before, during and following the full COVID-19 lockdown (means are weighted by age/sex/day of week, and to account for the underrepresentation of ages 65+ in the 2020 waves).

The vertical colored stacked bars show the average minutes per day in nine groups of activity/location/co-presence combinations ordered, bottom to top, by risk level. The left-hand column refers to the 2016 pre-lockdown pattern, the middle to late-May/early-June 2020 and the right to August 2020.

The behavioral changes associated with various stages of social restrictions can be clearly seen from this graphic. In particular, we see the shift from more to less risky combinations of activity/location/co-presence categories during full lockdown in May/June 2020, followed by a reversion (although not a complete reversal) in August 2020: the 71% of the day devoted to the lowest risk category (1) in 2016 increases to 88% of the day in May/June 2020, then decreases somewhat to 82% by late August 2020; and the 20% of the day spent in the highest risk category (5) in 2016 decreases to 7% in May-June 2020, increasing again to 11% in August. Changes in time spent in risk category 1 and risk category 5 across the 3 survey waves are indicated by lines connecting the 3 columns of Fig 1, showing the changing time spent in low risk activities (category 1) and high risk activities (category 5) across the 3 survey waves.

We used regression analyses of the (weighted) mean time spent in these high and low risk categories (1 and 5) across the three survey waves to provide an indication of the statistical significance of the differences between them. Two models were run, one for each risk category. May-June 2020 (full lockdown) was the reference category. 95% confidence intervals (CI) based on robust clustered standard errors clearly indicate that the differences in mean time across survey waves—associated with the movement into lockdown, and the reverse movements out of lockdown to intermediate levels of high- and low-risk behavior—are highly unlikely to be due to chance alone. Mean minutes spent in risk level 5 were 190 minutes higher (CI: 165–215, P < .001) in 2016 than during lockdown in May-June 2020, and 66 minutes higher (CI: 39–92, P < .001) during the following period of social restrictions in August 2020. Conversely, mean minutes spent in risk level 1 were 242 minutes lower (CI: -210 to -273, P < .001) in 2016 than during lockdown in May-June 2020, and 86 minutes lower (CI: -55 to -119, P < .001) during the following period of social restrictions in August 2020.

Describing the activity/location/copresence combinations that make up risk categories 1–5 in Fig 1 in more detail, at the bottom of the stacked columns the average daily time spent at home alone or with other household members doing self-care activities (including sleep, personal care, and meals) increased from 609 minutes/day in 2016 to 674 minutes/day in May-June 2020, then decreased to 632 minutes/day in August 2020. Unpaid work tasks done at home (cooking, cleaning, child-care, household maintenance), increased from 100 to 138 minutes during full lockdown, then decreased to 122 minutes following the easing of restrictions, while home leisure activities increased from 267 minutes/day in 2016 to 330 in full lockdown, then returning to 308 minutes/day by August. Paid home working increased dramatically from 42 to 116 minutes/day at full lockdown, but remained almost at the same level following the relaxation of social restrictions, at 111 minutes/day by August 2020. As we move up the column, risk category 2 includes activities such as leisure done away from home, but alone or with household members in open spaces (67 minutes/day to 43 at full lockdown, then back up again to 53 minutes/day), while risk category 3 includes activities such as leisure or caring done away from home in closed spaces, alone or with other household members (declining from 34 to 24, then back up again to 30 minutes/day). Risk category 4 comprises at home activities done with others not from the same household (22 to 7 minutes/day at full lockdown, rising back to a midway point at 13 minutes/day in August 2020). The highest risk category (level 5) includes activities such as leisure or care-giving done away from home with others not from the same household, and all paid work done at the workplace. In the case of the former there is a substantial decline from 126 to 48 minutes/day, followed by a return to 80 minutes/day by late August. In the case of paid work in the workplace there is an even more dramatic decline from 159 to 48 minutes/day between 2016 and full lockdown, with a similar rise following the easing of social restrictions back up to 81 minutes/day by late August.

Participation rates changed in a very similar way to average minutes/day (results available on request). The percentage engaging in the least risky (category 1) activities on an average day increased between 2016 and May/June 2020, then subsequently decreased (although not back to pre-pandemic 2016 levels). Moving up the categories of risk, the more-risky activities showed declining rates of participation to May-June 2020, then an increase again (although not a reversal to 2016 levels) by August 2020.

Populations’ time use patterns change, in normal times, only slowly [21]. So despite the 4-year distance from the earliest observation window, the relatively large changes in behavior using the same instrument and research panel between the baseline 2016 and May-June 2020 surveys may reasonably be interpreted as arising mainly from changes resulting from the imposition of full lockdown regulations in March 2020, while the changes evident in the short time-frame between May-June 2020 and August 2020 are likely to be directly related to the relaxation of social restrictions in July 2020. The evidence on changing patterns of behavior found in this data accords both with the assumptions about behavioral change made by those responsible for social restrictions policy, and with associated changes observed in national infection rates.

## Discussion

Recognizing that the risk of infection by COVID-19 is unlikely to disappear in the immediate future, it is likely that governments will need to continue to impose social restrictions on behavior (whether locally or nationally). In these circumstances policy-makers need to be provided with tools to be able to assess and quantify the sorts of changes in the risk-related behaviors that follow from these restrictions, and the impact they might be having on behavioral risk levels. The three CaDDI survey waves using the same instrument and research panel provide a new perspective on behavioral changes in daily life as it relates to the risk of infectious transmission. Our data document a shift from more to less risky daily behaviors (combinations of activity/location/co-presence categories) between the pre-pandemic pattern and full lockdown in May/June 2020, followed by a reversion (although not a complete reversal) of those patterns in August 2020, following the end of the first lockdown. The 20% of the day spent in the highest risk category in 2016 decreased to just 7% in May-June 2020, increasing again to 11% in August. Conversely, the 71% of the day devoted to the lowest risk category in 2016 increased to 88% of the day in May/June 2020, then decreased somewhat to 82% by late August 2020. Both these differences were found to be strongly statistically significant.

While the significant decrease in high-risk behavior recorded between pre-pandemic 2016 and May-June 2020, and the corresponding increase in home-based low-risk behavior, is likely to be attributable to the effects of the imposition of lockdown regulations in the UK in March 2020, the subsequent increase in high-risk behavior evident between the May-June and August 2020 survey waves (i.e. over only 2 months) is highly likely to be due to the relaxation of social restrictions in July 2020. The changing pattern of daily behavior associated with new phases of the COVID-19 social regulation process provides policy-makers with an overview of real-time behavioral changes associated with changing social restrictions, and will enable assessment of the efficacy of different restrictions in terms of changing risk-related behavior patterns.

Although to date time use diary surveys have not usually collected direct information on physical contact, which is a limitation of our data, the continuous and comprehensive behavioral data recorded in such diaries can be used to complement contact-based instruments. To this end, in future research we intend to validate our estimates of behavioral risk using empirical evidence from the CTUR’s collection of nearly 200 days of combined diary, body-camera and accelerometer evidence [22]. This evidence, (with photographs at approximately 45 second intervals throughout the diary day) allows direct observation of the physical proximity of the diarists to others in different combinations of activity.

A further limitation is that the CaDDI data is not strictly speaking a representative sample (being quota-based). Nonetheless due to the sampling design (using existing research panel members), once nationally representative quotas of respondents by age, sex, region and social group in 2016 were selected for each wave there was virtually no non-response. Other online questionnaire-based surveys (which did not collect time use diary data) conducted during the pandemic have suffered from relatively high levels of non-response–where this can be estimated—demanding considerable effort in the construction of non-response weightings to achieve representativeness [10].

There are a range of potential future opportunities arising from this data. As with most time-use diary surveys, the CaDDI is accompanied by an individual questionnaire, enabling detailed disaggregated analysis by socio-economic and demographic characteristics to explore the distributional correlates and consequences of daily activity patterns. So beyond population average measurement of the changing risk-related behavior in response to social restrictions, the questionnaire information also allows analysis of the distribution of the behavioral risk of social distancing measures across subgroups disaggregated by, for example, age [3], gender [23] and occupation [24]. This will enable the investigation of how different subgroups (for example, women compared to men, younger ages compared to older ages) respond behaviorally to changing regulations, enabling more efficient targeting of particular at-risk subgroups.

Secondly, the CaDDI diary also includes an instantaneous “how much did you enjoy the activity?” diary field, which can be used, in conjunction with the questionnaire information on respondents’ physical and mental health and wellbeing (GHQ12), to explore the subjective consequences of lockdown; for example enabling the identification of vulnerable groups with lower levels of wellbeing during the stresses imposed by the COVID-19 virus, and whether this affects their behavioral response to social restrictions.

Finally, there are many larger-scale time-use diary datasets, designed and collected by National Statistical Institutes, which may be used for validation purposes, and which also provide the potential for both cross-national and historical comparisons [25]. While the traditional large-scale survey design of these surveys precludes rapid deployment during pandemic conditions, the CaDDI survey in 2016 provided similar information from 8 other countries (including the USA), opening the opportunity for future cross-national pre- and post-COVID-19 research along similar lines in other countries.

## Supporting information

S1 Table Lookup table for the nine aggregated activity/copresence/location categories shown in Fig 1. (DOCX)
