# Supplementary material for: A new perspective from time use research on the effects of social restrictions on COVID-19 behavioral infection risk
Source: PLoS One. 2021 Feb 10;16(2):e0245551. doi: 10.1371/journal.pone.0245551 (PMC7875376; doi:10.1371/journal.pone.0245551)
Supplement: S1 Table — (DOCX) [file pone.0245551.s001.docx]

**S1 Table. Lookup table for the nine aggregated activity/copresence/location categories shown in Figure 1**

|  |  | risk levels | Including these cells in assignment table |
| --- | --- | --- | --- |
| 1 | Personal care, alone or with HH member, at home | 1 | 1.0 2.0 3.0 4.0 |
| 2 | Unpaid work, alone or with HH member, at home | 1 | 5.0 6.0 7.0 8.0 21.0 23.0 |
| 3 | Leisure, alone or with HH member, at home | 1 | 27.0 28.0 29.0 32.0 33.0 34.0 36.0 |
| 4 | Paid work, study, prayer, alone/HH member, at home | 1 | 26.0 10.0 17.0 18.0 19.0 20.0 25.0 |
| 5 | Leisure or caring, alone or with HH member, away, open air/equivalent | 2 | 1.2 2.2 3.2 5.2 6.2 7.2 8.2 11.2 12.2 31.2 13.2 25.3 |
| 6 | Leisure/caring, alone/with HH member, away, enclosed space | 3 | 21.2 23.2 27.2 28.2 29.2 32.2 33.2 34.2 36.2 4.2 22.2 24.2 9.0 9.2 20.2 11.3 12.3 31.3 |
| 7 | Leisure/caring or paid work, with non-HH member, at home | 4 | 1.1 2.1 3.1 4.1 5.1 6.1 7.1 8.1 9.1 21.1 23.1 27.1 28.1 29.1 32.1 33.1 34.1 36.1 26.1 10.1 17.1 18.1 19.1 20.1 25.1 22.1 24.1 |
| 8 | Leisure/caring, with non-HH member, away from home | 5 | 1.3 2.3 3.3 5.3 6.3 7.3 8.3 21.3 23.3 27.3 28.3 29.3 32.3 33.3 30.3 35.3 34.3 36.3 14.3 15.3 30.3 35.3 34.3 36.3 14.3 15.3 16.3 30.3 35.3 4.3 22.3 24.3 9.3 26.3 10.3 19.3 20.3 |
| 9 | Paid work or study, any copresence, away from home | 5 | 17.3 18.3 19.3 20.3 25.3 14.3 15.3 |

**Notes:** Table S1 provides a lookup table for replication purposes setting out the detailed composition of the nine activity/copresence/location categories of Figure 1, based on the activity number and column number information shown in Table 2. For example Category 1, low risk activities in the “Personal care, alone or with HH member, at home” activity group is comprised of four cell references (activity number + column number): 1.0 (for activity 1; time spent asleep, and column 0; at home, alone or with other household members); plus cell reference 2.0 (resting time, again at home, alone or with other household members) plus cell 3.0…. and so on.
